# Hydrodynamic and Hemodynamic Interactions in Chronic Hydrocephalus

**DOI:** 10.3390/biomedicines11112931

**Published:** 2023-10-30

**Authors:** Cyrille Capel, Kimi Owashi, Johann Peltier, Olivier Balédent

**Affiliations:** 1 Department of Neurosurgery, Hospital University Center of Amiens-Picardie, 80054 Amiens, France; peltier.johann@chu-amiens.fr; 2 CHIMERE UR UPJV 7516, Jules Verne University, 80000 Amiens, France; kimi.owashi@u-picardie.fr (K.O.); olivier.baledent@chu-amiens.fr (O.B.); 3 Image Processing Department, Hospital University Center of Amiens-Picardie, 80054 Amiens, France

**Keywords:** normal pressure hydrocephalus, Hakim’s disease, cine-MRI, hydrodynamic, fluid dynamic, cerebrospinal fluid

## Abstract

Background: During a cardiac cycle, intracranial pressure is related to arterial entry into the cranium and its interaction with intracranial compliance. The arterial inflow is compensated by intracranial compliance and, initially, the flushing of cerebrospinal fluid (CSF) into the cervical subarachnoid spaces. Our objective is to analyze the interactions between intracranial arteriovenous exchange and cerebrospinal fluid oscillations. Method: A total of 23 patients (73 ± 8 years) with suspected chronic hydrocephalus (CH) underwent an infusion test and phase-contrast MRI. Rout is an important factor in the diagnosis of CH. Patients were divided into 2 populations: *probableCH* (Rout: resistance to CSF outflow) (Rout > 12 mmHg/mL/min, 13 patients) and *unlikelyCH* (Rout < 12 mmHg/mL/min, 10 patients). We measured the intracranial vascular volume (arteriovenous stroke volume: SV_vasc_) and CSF (CSF stroke volume at upper cervical level: SV_CSF_) volume variations during the cardiac cycle. Results: In the whole population, we observed a significant correlation between SV_vasc_ and SV_CSF_ (R^2^ = 0.43; *p* = 0.0007). In the population *unlikelyCH*, this correlation was significant (R^2^ = 0.76; *p* = 0.001). In the population *probableCH*, this correlation was not significant (R^2^ = 0.17, *p* = 0.16). Conclusions: These results show that the link between the compliance of the oscillating CSF and the abrupt arterial inflow seems to be altered in CH. CSF oscillations between intracranial and cervical fluid spaces limit the impact of the abrupt arterial inflow.

## 1. Introduction

Diagnosing chronic hydrocephalus (CH) is a challenging task. Currently, the most widely accepted definition involves the presence of ventricular dilation in association with neurological disorders that are either completely or partially reversible after cerebrospinal fluid (CSF) diversion surgery [1]. In CH, intracranial pressure is usually not elevated [1] but there is a disturbance of intracranial-pressure-derived parameters assessed through an infusion study [2]. Infusion studies in CH patients have revealed the presence of slow B-waves, an increase in resistance to CSF outflow (Rout), and correlations between the amplitude of intracranial pressure and intracranial pressure (RAP) [2,3]. The diagnosis of CH patients via infusion studies is considered one of the most crucial assessments for determining which patients require a shunt procedure and which do not. The positive predictive value of Rout is nearly 80% when it exceeds 12 mmHg/mL/min [4,5]. Rout is frequently used to differentiate between patients with probable CH and those with unlikely CH, aiding in surgical decision making.

Intracranial pressure refers to the pressure of cerebrospinal fluid (CSF), and its generation is a result of the interplay among CSF, arterial, and venous flows. This interaction is traditionally governed by the Monro–Kellie doctrine [6]. It states that the intracranial volume is constant. There are four intracranial compartments: cerebral parenchyma, arterial blood, venous blood, and CSF. Any changes in the volume of one compartment must be compensated by the others, with CSF playing a key role. Recently, this doctrine, initially conceptualized by Cushing [7], has been revisited by Wilson [8]. In this Monro–Kellie 2.0, Wilson presents the importance of the venous compartment in the regulation of intracranial pressure. Reciprocally, an alteration in the venous drainage disturbs intracranial pressure. There are intricate interactions among arterial blood flow, venous blood flow, and CSF flow in the regulation of intracranial pressure.

There is both a pulsatile and slow circulation of CSF, with each type governed by different physiological and physical laws. During a long period, the Monro–Kellie doctrine is crucial. Indeed, a disorder in the volume of a compartment needs to be compensated by the others to preserve the mean intracranial pressure. During the cardiac cycle, the application of the Monro–Kellie doctrine is not appropriate, suggesting its inadequacy in certain scenarios. The intracranial pressure pulse waveform is not constant, changing with both the respiratory and cardiac cycles. The pulsatility of intracranial pressure is well documented [2,9,10]. Within one cardiac cycle, changes in intracranial pressure are primarily attributed to CSF and the flow of arterial and venous blood into the craniospinal system [11,12]. During this cycle, there are variations in intracranial vascular volume due to arterial blood inflow during a systole. This increase in vascular volume is not immediately compensated by venous blood outflow, leading to an arteriovenous delay [11,13]. CSF flows passively through the foramen magnum from intracranial subarachnoid spaces to spinal subarachnoid spaces during the systolic phase. This flow partially compensates for the increase in intracranial vascular volume and regulates intracranial pressure. During the diastolic phase, the venous compartment compensates for the decrease in intracranial vascular volume. This results in CSF flowing from spinal subarachnoid spaces to intracranial subarachnoid spaces. Together, these phenomena lead to variations in intracranial volume over the course of one cardiac cycle [14]. These volume variations play a major role in the genesis of intracranial pressure.

Phase-contrast MRI (PCMRI) is a unique tool to non-invasively evaluate this pulsatile circulation of blood and CSF during a cardiac cycle [15,16,17,18]. In CH, several studies have utilized intraventricular CSF pulsatility measurements through the Sylvius aqueduct to assess CSF stroke volume. However, the interpretation of these findings is still a subject of debate. Initially, this approach was motivated by the observation of frequent flow artifacts, known as flow-voids, in the ventricles of many CH patients during the 1990s [19,20]. Subsequently, studies reported a strong correlation between the CSF stroke volume in the aqueduct and postoperative improvements in CH patients [20,21,22,23]. However, other studies have indicated that intraventricular CSF pulsatility is more associated with ventricular dilation rather than the underlying pathology [24]. Another approach was more global and proposed the investigation of all the intraventricular, intracranial, and spinal CSF compartments but also arterial and venous flows. CSF oscillations respond to variations in intracranial pressure, which are generated by arteriovenous interactions, and thus to the intracranial vascular volume changes during one cardiac cycle [14]. It seems relevant to analyze arteriovenous and CSF oscillations in CH [16,25]. 

Our objective is to analyze arterial blood, venous blood, and CSF oscillations and interactions using PCMRI in CH patients and patients that present a passive ventricular dilation. 

## 2. Patients and Methods

### 2.1. Patients

We included 23 prospective patients (73 ± 8 years) from the REVERT project (revertproject.org). All of these patients presented suspected CH. They underwent morphological MRI with PCMRI acquisitions. The hydrocephalus of all patients was communicating after neuroradiological morphologic analysis.

After MRI, patients underwent a lumbar infusion test. The delay between PCMRI and infusion test was 2 months or less. A senior neurosurgeon performed an infusion test in patients with suspected CH in the lateral decubitus. An 18-gauge needle was inserted into the lumbar intrathecal space and connected to a pressure transducer and syringe pump via a 3-way tap.

Intracranial pressure was analyzed using ICM+^®^ software (version 9.1.0.17) (icmplus.neurosurg.cam.ac.uk (accessed on 16 march 2023)). Baseline intracranial pressure (Pbaseline) was measured for 15 to 20 min. Saline was then injected at a rate of 1.5 mL/min until plateau pressure (Pplateau) was reached. Intracranial pressure was analyzed using ICM+^®^ (icmplus.neurosurg.cam.ac.uk). Rout was calculated via this by using the formula $Rout=\frac{\left(Pplateau-Pbaseline\right)}{Q}$ (Q: infusion rate).

Patients were divided in two groups: *probableCH* if Rout > 12 mmHg/mL/min (13 patients) and *unlikelyCH* if Rout < 12 mmHg/mL/min (10 patients). 

### 2.2. MRI Acquisition

All included patients underwent morphological analysis through MRI and the dynamic study of blood and CSF flows through PCMRI. 

**Figure 1:**
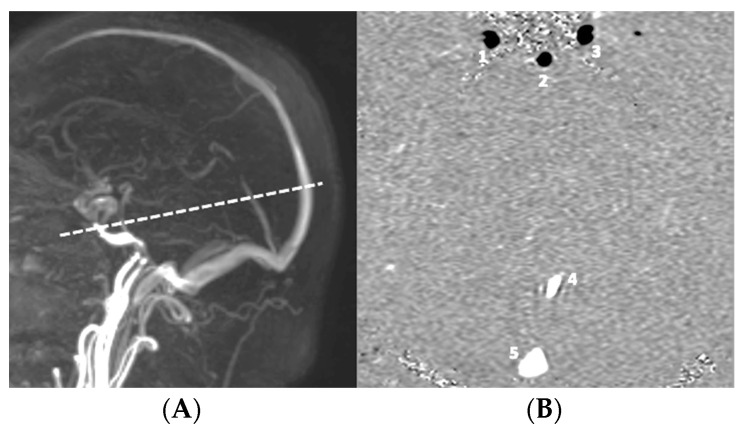
Slice plane position for vascular acquisition of phase-contrast MRI for hemodynamic evaluation. (**A**) Position of a 3D time-of-flight sequence; (**B**) axial slice of phase-contrast MRI (encoding velocity = 60 cm/s) through 1—right carotid artery; 2—basilar artery; 3—left carotid artery; 4—straight sinus; 5—superior sagittal sinus.

**Figure 2:**
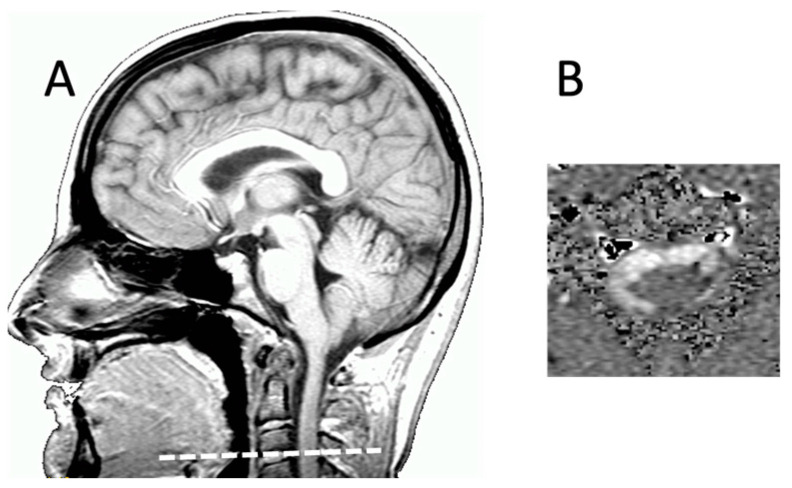
Slice plane position for acquisition of phase-contrast MRI for hydrodynamic evaluation. (**A**) Slice position on sagittal T1 acquisition through the C2C3 intervertebral disc and perpendicular to the assumed direction of circulation of CSF into the spinal subarachnoid spaces; (**B**) axial slice of phase-contrast MRI through the subarachnoid spaces at the level of the C2C3 intervertebral disc (encoding velocity = 5 cm/s) for analysis of CSF dynamics. This image represents 1 of the 32 phases studied during one cardiac cycle, with the subarachnoid spaces appearing like a white crown due to the caudocranial circulation of CSF. At other phases, it appears in black if CSF flows according a craniocaudal direction during CSF oscillation.

Planes of slices were placed perpendicularly to the presumed flow direction of the studying compartment. Vascular analysis was based on a plane upstream of the Willis circle (Figure 1). CSF flow analysis was based on an acquisition through the upper cervical SAS (C2C3level) (Figure 2).

Sequence parameters are presented in Table 1. Encoding velocity was 60 cm/s for vascular study and 5 cm/s for CSF analysis. Each sequence lasted about a minute.

### 2.3. Data Analysis

Data were analyzed using in-house software (Flow 2.0—march 2021) for the semi-automatic delineation of the blood vessels and CSF regions [11]. Velocities and segmented areas were calculated through each of the 32 cardiac phases to produce the flow curves during the cardiac cycle. Stroke volumes were defined as the average of the cranio-caudal and caudo-cranial volumes displaced through the region of interest during the cardiac cycle [26]. 

For CSF analysis, the curve of flow evolution over the cardiac cycle was determined. We secondarily integrated this curve. One part was negative and the other was positive. We averaged the absolute value to obtain the stroke volume of the CSF measured at the cervical level. This is the volume displaced through this cross-sectional plane, characterizing the variation in intracranial CSF volume during a cardiac cycle [11,14].

Cerebral arterial blood flow (CBF_a_) was defined as the sum of the flow through the internal carotid and basilar artery. Cerebral venous blood flow (CBFv) was measured as the sum of the flow of the superior sagittal sinus and straight sinus. As CBFv does not drain all the CBF itself (other peripheral veins are involved, such as epidurals), a venous correction factor (α factor) was calculated as equal to the mean(CBFa)/mean(CBFv). It was thus possible to provide a theorical *_corrected_*CBFv flow curve = α factor * CBFv flow. When this venous factor is equal to 1, the *_corrected_*CBF_v_ is equal to the CBF_v_. This corresponds to exclusive jugular venous drainage. When the correction factor is >1, it shows the magnitude of accessory drainage paths.

To evaluate how the cerebral blood volume increases during the cardiac cycle, the cerebral arteriovenous flow curve was calculated through subtraction of the CBF_a_ and *_corrected_*CBF_v_. The arteriovenous stroke volume was calculated as the CSF stroke volume. 

The studying parameters were:

- SV_CSF_: corresponds to the stroke volume of CSF, which oscillates through the upper cervical SAS at the level of the C2C3 intervertebral disc during one cardiac cycle. This reflects variations in intracranial CSF volume during a cardiac cycle.
- SV_AV_: corresponds to the volume of blood, which oscillates in the cranial compartment during one cardiac cycle. This reflects variations in intracranial vascular volume during a cardiac cycle.
- Hemohydrodynamic ratio: ratio between SV_CSF_ and SV_AV_. SV_CSF_ is the passive response of CSF to a pressure gradient between intracranial and spinal SAS (ΔP). SV_AV_ is the main factor in intracranial volume variation during a cardiac cycle (ΔV). The combination of these two parameters gives the ratio of a change in volume to a change in pressure.

### 2.4. Statistical Analysis

Student’s *t*-test was used to compare hemodynamic parameters between patients with *probableCH* and patients with *unlikelyCH*. The threshold of significance was set to a *p*-value of 0.05.

Correlation study was performed using a Pearson correlation and linear regression. The threshold of significance was set to a *p*-value of 0.05.

### 2.5. Ethics

All these patients came from the REVERT project research protocol (revertpro-ject.org). This project has been approved by our ethics committee (ID-RCB: 2021-A00240-41).

## 3. Results

### 3.1. Comparison of Hemodynamic and Hydrodynamic Parameters in Probable and Unlikely Hydrocephalus Populations

We found no difference in CSF and arteriovenous stroke volumes in our two populations. SV_AV_ and SV_CSF_ were not different between *unlikelyCH* and *probableCH* populations. All results are presented in Table 2.

### 3.2. Analysis of Hemohydrodynamic Interactions in Probable and Unlikely Hydrocephalus Populations

**Figure 3:**
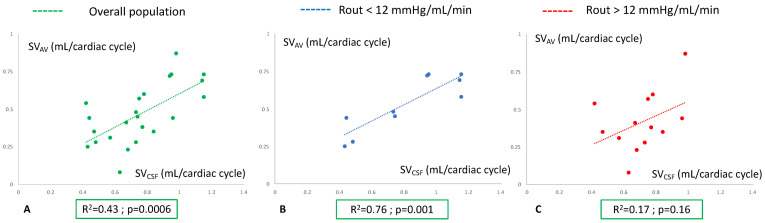
Analysis of arteriovenous and cerebrospinal fluid (CSF) interactions through a correlation study. (**A**) Study of the correlation of the arteriovenous stroke volume (SV_AV_) and the cervical CSF stroke volume (SV_CSF_) in the overall population. (**B**) Study of the correlation of the SV_AV_ and SV_CSF_ in the unlikely chronic hydrocephalus population (resistance to CSF outflow below 12 mmHg/mL/min). (**C**) Study of the correlation of the SV_AV_ and SV_CSF_ in the probable chronic hydrocephalus population (resistance to CSF outflow higher than 12 mmHg/mL/min). Statistic test: Pearson’s correlation.

In the overall populations of suspected hydrocephalus, we found a significant correlation between SV_AV_ and SV_CSF_ (R^2^ = 0.43; *p* = 0.0006) (Figure 3A). In the *unlikelyCH* population, the correlation between SV_AV_ and SV_CSF_ remained significant (R^2^ = 0.76; *p* = 0.001) (Figure 3B). In the *probableCH* population, a correlation between SV_AV_ and SV_CSF_ was not present (R^2^ = 0.17; *p* = 0.16) (Figure 3C).

The hemohydrodynamic ratio in *unlikelyCH* was 1.55 ± 0.25 and that in *probableCH* was 2.13 ± 1.71. This index was not different between our two populations (*p* = 0.03).

## 4. Discussion

Arteriovenous and CSF interactions can be analyzed using PCMRI. However, the response of CSF to arteriovenous changes during the cardiac cycle differs between CH and non-CH patients. This reflects an inappropriate response of CSF to changes in intracranial vascular volume, thus indicating a disturbance in intracranial pressure.

### 4.1. Comparison of Hemodynamics in Probable and Unlikely Hydrocephalus Populations

SV_AV_ was not different between *unlikelyCH* and *probableCH* populations. Balédent et al. [16] studied the hemodynamics and craniospinal hydrodynamics in a CH population and an aging control group. A decrease in arterial cerebral blood flow was found. Another study showed a decrease in periventricular cerebral blood flow in hydrocephalus patients [27]. Arteriovenous and CSF interactions were different between CH patients and the control group [16]. Temporal analysis of arterial and venous flows showed a precocious venous systolic peak in hydrocephalus [13,16]. A venous compensatory mechanism to the cerebral compliance disorders was proposed. Both arterial and venous pulsatility were altered in CH, but these alterations were compensated for by a temporal adaptation of venous pulsatility, preserving the overall arteriovenous pulsatility and variations in intracranial vascular volume, as observed in our results.

### 4.2. Comparison of Hydrodynamics in Probable and Unlikely Hydrocephalus Populations

SV_CSF_ was not significantly different between *unlikelyCH* and *probableCH* populations. This result is in line with previous studies [16]. They have reported a decrease in intracranial SAS CSF pulsatility and a preservation of cervical CSF pulsatility. Intraventricular CSF pulsatility became preponderant in the intracranial compartment [28], possibly indicating an alteration of intracranial SAS compliance [16,29,30]. Intraventricular compliance would then be recruited, as evidenced by the increased pulsatility of intraventricular CSF. Temporal analysis of CSF flows supports this observation, revealing a precocious systolic peak of CSF in CH patients compared to controls [16]. 

However, analyzing CSF alone may not provide diagnostics or pathophysiological insights. CSF has a passive circulation and responds to variations in intracranial pressure, which is influenced by vascular volume changes and the biomechanical properties of the brain. The intracranial pressure waveform is primarily determined by intracranial vascular and CSF volume variations [31], highlighting the importance of the combined analysis of CSF and arteriovenous data.

### 4.3. Analysis of Hemohydrodynamic Interactions in the Two Patient Groups

We found a correlation between SV_AV_ and SV_CSF_ in the overall population. This causes a response of CSF to the intracranial vascular volume variations, which generate intracranial pressure [14,31]. Intracranial pressure variations are also due to other potential compensatory mechanisms, which are not currently well known. It seems that the cranium skull presents a little deformability. Indeed, intracranial pressure can be measured using a non-invasive transcranial measurement [32]. Other compensatory mechanisms could potentially be the brain parenchyma, which could present an intrinsic compliance due to its elastic properties [33]. 

These assumptions can explain the existence of different responses of the CSF function of these alternative compensatory mechanisms to preserve intracranial pressure. A correlation between SV_AV_ and SV_CSF_ is present in the *unlikelyCH* population but is not present in the *probableCH* population. For similar changes in intracranial vascular volume, the hydrodynamic response is different in our two populations. This could be linked to other compensatory mechanisms of intracranial pressure. But these remain unknown.

### 4.4. Compliance in Chronic Hydrocephalus

The analysis of the hemohydrodynamic ratio shows that SV_CSF_ is mainly reduced in front of SV_AV_. In a few cases, we observed a very high SV_CSF_ in front of SV_AV_. The hemohydrodynamic ratio is not useful for CH diagnostics. There is an important heterogeneity in the hydrodynamics in CH. An increase in SV_CSF_ relative to SV_AV_ suggests an alteration in intracranial compliance, whereas a decrease indicates a spinal compliance alteration. Both of these hydrodynamic patterns are associated with a clinical history of CH, reflecting different underlying pathophysiologies. The classification of chronic hydrocephalus may need to be revisited based on these distinct hydrodynamic behaviors and suspected alterations.

**Figure 4:**
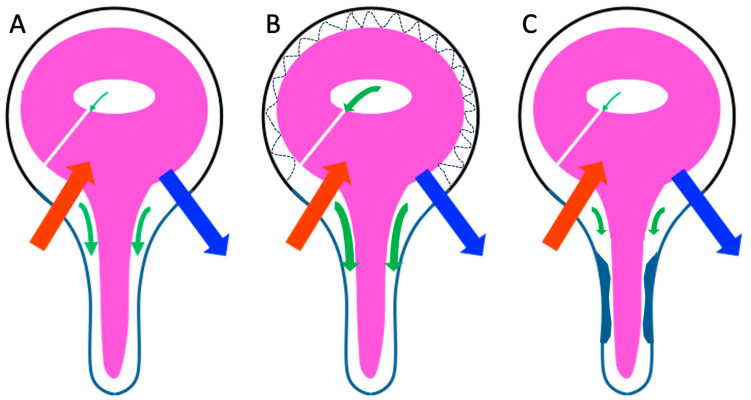
Hemohydrodynamic interactions in chronic hydrocephalus. (**A**) In populations without chronic hydrocephalus (CH) (control or unlikely CH patients), each cardiac cycle produces a variation in intracranial vascular volume due to a delay between arterial (red arrow) and venous (blue arrow) systoles. CSF partially but instantaneously compensates for this variation in intracranial vascular volume by flushing it from the intracranial subarachnoid spaces (SASs) into the spinal SAS (green arrow). (**B**) In patients with felted intracranial SAS, there is a loss of intracranial SAS compliance. This causes increased flushing of the CSF into the spinal SAS. (**C**) In CH patients with impaired compliance of spinal SAS (as may be observed in anatomically restricted spinal SAS), there is a reduction in CSF flushing to these SAS.

Craniospinal compliance cannot be considered as a single entity. In fact, intracranial and spinal compliance separately exist on the scale of the cardiac cycle. Intracranial compliance (Figure 4) is the result of CSF pulsatility at the SAS and intraventricular level, as well as hemodynamic adaptations, notably venous.

### 4.5. Limits

Our study is limited in terms of the number of patients. It seems necessary to increase our populations in order to better discriminate the different hydrodynamic alterations predicted in the *probableCH* group. Indeed, a larger number of patients would enable us to better observe and understand the heterogeneity of results concerning alterations in CSF pulsatility. This would enable us to approach disorders associated with altered intracranial or spinal compliance.

Our groups are based on the analysis of intracranial pressure and CSF flow resistance. However, this parameter has a low negative predictive value [3]. A future study should propose an analysis based on the effectiveness of surgery. This remains the only diagnostic proof of chronic hydrocephalus. However, according to recent studies, the effectiveness of surgery depends on how long symptoms have been present [34,35]. Late treatment may not be effective. This major factor therefore needs to be taken into account in patient selection. In addition, there is a change in cerebral hydrodynamics from a volumetric point of view through PCMRI [36] or from a barometric point of view during an infusion test [37].

## 5. Conclusions

The hydrodynamic response to the intracranial hemodynamic constraints is disturbed in CH. There are multiple hydrodynamic responses with a hyperdynamic CSF in some cases and an hypodynamic CSF in others. These alterations are different but link to CH development. Hemohydrodynamic analysis seems to be essential to understand neurofluid flows in CH, which is not an entity but an association of multiple pathologies. These different alterations are probably linked to different alterations in compliance. Compliance is an emerging concept in CSF research. This concept needs to be precisely defined. Indeed, there are many anatomic sites that regulate craniospinal compliance. In addition, it is necessary to determine the behavior of the different temporal scales of analysis: the supracardiac scale, where Monro–Kellie’s law can be applied, or the cardiac cycle scale, where Monro–Kellie’s law cannot be applied.

## Figures and Tables

**Table 1:** Acquisition parameters of phase-contrast MRI.

	CINE-PC
Venc (cm/s)	60 (blood)/5 (CSF)
FOV (cm^2^)	14 × 14
Resolution (mm^2^)	1 × 1
Thickness (mm^2^)	2
Flip Angle (degree)	30
EPI-factor	NA
SENSE	1.5
TE (ms)	6.6
TR (ms)	10.9
Readout time (ms)	5.3
Number of images	32
Acquisition time (s)	50–115
Number of images per cycle	32

**Table 2:** Comparison of hemodynamic and hydrodynamic parameters in unlikely and probable hydrocephalus groups. *cc: cardiac cycle*.

	Unlikely Hydrocephalus	Probable Hydrocephalus	*p*-Value
Age (years)	75 ± 7	73 ± 8	0.63
Sex ratio (F/M)	2/3	6/7	0.82
SV_AV_ (mL/cc)	0.82 ± 0.20	0.71 ± 0.17	0.44
SV_CSF_ (mL/cc)	0.54 ± 0.18	0.42 ± 0.20	0.13
